# Based on Regular Expression Matching of Evaluation of the Task Performance in WSN: A Queue Theory Approach

**DOI:** 10.1155/2014/654974

**Published:** 2014-10-23

**Authors:** Jie Wang, Kai Cui, Kuanjiu Zhou, Yanshuo Yu

**Affiliations:** School of Software Technology, Dalian University of Technology, Dalian 116620, China

## Abstract

Due to the limited resources of wireless sensor network, low efficiency of real-time communication scheduling, poor safety defects, and so forth, a queuing performance evaluation approach based on regular expression match is proposed, which is a method that consists of matching preprocessing phase, validation phase, and queuing model of performance evaluation phase. Firstly, the subset of related sequence is generated in preprocessing phase, guiding the validation phase distributed matching. Secondly, in the validation phase, the subset of features clustering, the compressed matching table is more convenient for distributed parallel matching. Finally, based on the queuing model, the sensor networks of task scheduling dynamic performance are evaluated. Experiments show that our approach ensures accurate matching and computational efficiency of more than 70%; it not only effectively detects data packets and access control, but also uses queuing method to determine the parameters of task scheduling in wireless sensor networks. The method for medium scale or large scale distributed wireless node has a good applicability.

## 1. Introduction

Most wireless sensor network (WSN) missions are to detect and environmental reporting events. Since wireless sensor networks usually work under severe environments, their performance is often difficult or impossible to assess accurately. Therefore, how to evaluate the performance in the wireless sensor network for task communication is the research emphasis in recent years, which becomes one of the most attractive.

Most current researches have focused on how to provide authentication, confidentiality, integrity, nonrepudiation, and access control ad hoc [1, 2]. Distributed authentication is a very common approach to solve ad hoc security issues [3, 4]. However, highly secure ad hoc approach in certain circumstances the lack of a common approach [5, 6].

Because regular expressions provide excellent communication skills and flexibility, they have been widely used in a variety of network security applications, such as antivirus scanning, network intrusion detection and prevention systems [7], firewalls, and traffic classification and monitoring [8]. Deterministic finite automata (DFA) and nondeterministic finite automata (NFA) are the typical application of regular expressions. But this requires a certain store or time-consuming cycle tolerable for resource-constrained wireless sensor condition is basically meeting the application requirements. Performance evaluation based on the communication task queuing model theory methods [9, 10], in recent years, published several class methods.

However, previous methods suffer from the following disadvantages. In typical queuing model, customer arrival and the service processing are independent. However, they are relative to the sensor communication scheduling tasks. Sensors cannot receive communication and run the state machine at the same time. Usually the sensor is constant communication the occupied and communication task processing footprint. The arrival of the communication task has different priorities. The high-priority tasks can preempt the low-priority tasks, and the low-level tasks continue to run after the high-priority tasks processing is complete. Time constraints: the traditional system analysis often considered the average time while the task's communication between wireless sensors needs to consider the maximum time after which the time state machine will transmit to another state.

The method of finite automata has researched in the regular expression matching system security for the wireless sensor networks, and matching performance is ignored to further discussion [11]; similarly, the evaluation of the performance based on the queue model in the wireless sensor network (WSN) has been discussed, but the system security matching method not to do more research [12]; therefore, we combined with the previous work, in this paper, the security matching method of finite automata, and the performance of the queuing model was discussed in WSN.

The rest of the paper is organized as follows.  Section 2 reviews regular expression two-stage matching strategy.  Section 3 explains the regular expression matching approach. In Section 4, model of task scheduling based on queuing theory is proposed. In Section 5, the priority queue with two classes of tasks is proposed. We describe the performance of the wireless sensor communication tasks based on queuing theory in Section 6. In Section 7, simulations are conducted for illustrating the performance of our scheme. Finally, we conclude this paper in Section 8.

## 2. Regular Expression Two-Stage Matching Strategy [11]

Recent research has paid much attention to reduction of the huge memory usage for DFA-based regular expression matching, as DFA is the preferred representation of regular expression matching. As a matter of fact, they can only achieve memory reduction for specific regular expression or signature sets of simple. High-speed regular expression matching for real-world signature sets that contain thousands of complex regular expressions can be hardly achieved. In modern networking devices, TCAMs (off-the-shelf chips) have been widely deployed. However, even if techniques such as D2FA [13, 14] are employed, tables of DFA and NFA are too big to be stored in TCAMs. In 2012, the RegexFilter (a high-speed and memory efficient technique) was presented by Liu et al. [15]. Regular expression matching was been sped up by quickly searching these regular expressions that may match each arriving item as little as possible. However, this method only cares about the profiteering stage and left the verifying stage without any optimization.

### 2.1. Profiteering Stage

**Figure 1:**
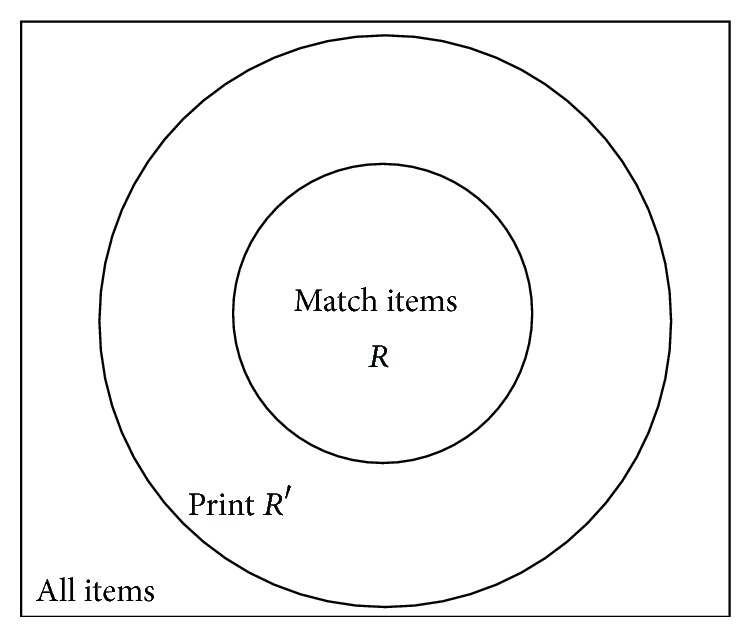
Relationship between profiteering stage set and match items.

For instance, there is a regular expression set called *R*; another set *R*′ is constructed so that any unmatched item of *R*′ is also an unmatched item of *R*. An item that does not match any regular expression in the set [15] is unmatched item of a regular expression set. Given an item *i*, it will match against *R*′ to get set *O*(*R*′; *i*) firstly. If *O*(*R*′; *i*) is empty, it does not obviously match any member in *R* and therefore this item can be skipped safely; otherwise, matching it against *T*  (*R*; *O*(*R*′; *i*)) will continue, where *O*(*R*; *i*)⊆*T*  (*R*; *O*(*R*′; *i*))⊆*R*.  Figure 1 shows the relationship between match items and print (*R*′). Because most items are unmatched and the match cost of *R*_0_ is much less than that of *R*, the overall throughput of this approach can be much higher than directly matching against *R*.

### 2.2. Verifying Stage

In the verifying stage, how to build correlation from profiteering print and reduce the memory cost of DFA tables is the main point that needs to be handled. A DFA is presented by a 5-tuple (*Q*; ∑; *δ*; *q*_0_; *A*) where *Q* is a set of states, *P* is an alphabet, ∑×*Q* → *Q* is the transition function, *Q*_0_ is the start state, and *A*⊆*Q* is a set of accepting states. The major part we should deal with is the DFA-based algorithms with the large amount of memory requirement to store the transition table. Software-based [16–18] and FPGA-based [19, 20] regular expression matching algorithms are traditional approaches with many shortcomings. TCAM-based solutions have the advantages of easy encoding and high parallelism [13]. Three novel techniques, transition sharing, table consolidation, and variable striding, were proposed by Liu et al. to reduce TCAM space and improve matching speed.

## 3. Regular Expression Matching Approach

**Figure 2:**
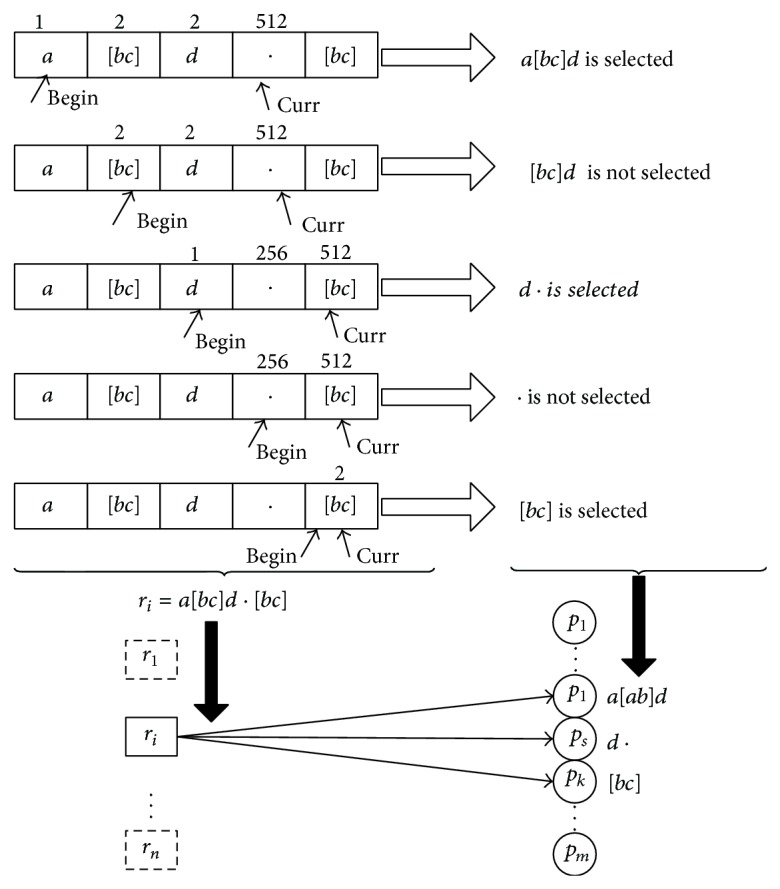
Process of generating print *p*_*i*_ from *r*_*i*_.

The selecting process of regular expression “*a*[*bc*]*d* : [*bc*]” with five atoms is shown in Figure 2. The parameter *β* = 256 is the boundary we define and the expression size of every print should be less than *β*. The selecting stage begins from the first atom. The curr pointer keeps moving to the next atom if ES(*r*) value of the regular expression print between the begin pointer and end pointer until the curr pointer arrives at the fourth atom “·”, ES(*a*[*bc*]*d*:) = 1∗2∗1∗256 = 512 > *β*. Condition ES(*r*) < *β* does not hold, and print *a*[*bc*]*d* is selected. Then a directed line from *r*_*i*_ to *P*_*i*_ to mark the correlation relationship is constructed. Then, in step 2, it is included in the already selected print “*a*[*bc*]*d*”, although [*bc*]*d* satisfies the condition. According to section A, *a*[*bc*]*d* has higher matching probability (MP) than [*bc*]*d*; thus, [*bc*]*d* is not selected. The same criteria are processed in steps 3, 4, and 5 to select print.

After selecting the print, a relationship of this graph called correlation sequence is generated as a directed graph from *r*_[1;…;*n*]_ to *p*_[1;…;*m*]_.

Every package will be transmitted across certain nodes *N*_1_; *N*_2_; …; *N*_*n*_ according to ad hoc wireless protocol. These nodes will be grouped into two groups: one group for profiteering stage and the other group for verifying stage. Extra package fields are adopted to make each node work collaboratively and communicate with the other.

**Figure 3:**
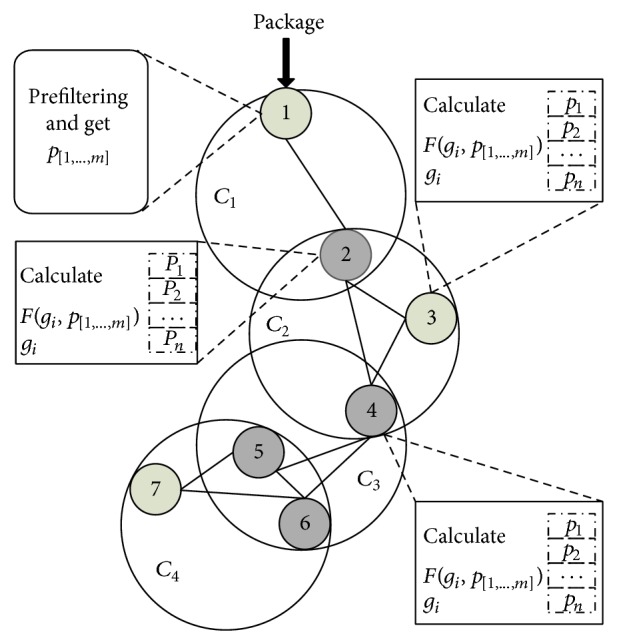
Matching process of ad hoc packages.

The package matching process is demonstrated in Figure 3. Taking the limited computing power of each wireless node into consideration, the calculation of *F*(*g*_*i*_; *p*_[1;…;*m*]_) is simplified to be addition only. At first, the feature vector *p*_*i*_ needs to be stored so that the sum of *p*_*i*_ can be calculated to get *F*(*g*_*i*_; *p*_[1;…;*m*]_). The profiteered correlation sequence *p*_[1⋯*m*]_ will be generated in node 2 after profiteering stage in node 1. Then, if *p*_[1⋯*m*]_ is not empty, the *F*(*g*_*i*_; *p*_[1;…;*m*]_) is calculated in node 2 by adding the feature vector *p*_*i*_. Verifying process will continue in node 2 using the group *g*_*i*_ in its memory when *F*(*g*_*i*_; *p*_[1;…;*m*]_) is larger than *ψ*. Otherwise, the package will be transmitted to the next hop and *g*_*j*_ will be matched continually.

## 4. Queue Model Description [12]

**Figure 4:**
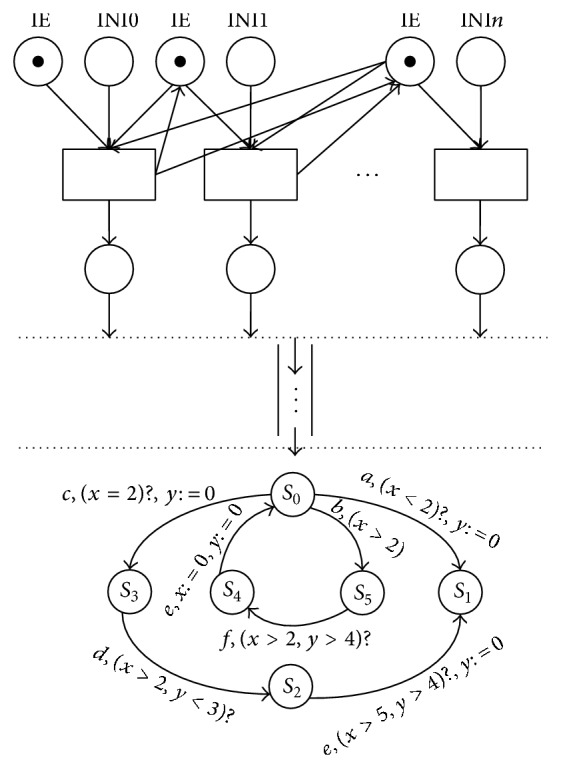
Queuing theory model of the wireless sensor network with *n* communication task's scheduling.

The pattern of communication between wireless sensors can be divided into two modes: the synchronous and the asynchronous modes. In synchronous mode, when a plurality of communication tasks are triggered, the tasks scheduling will be suspended. At this moment, the levels of query priority and processes priority are executed in sequence. This mode has a higher efficiency when the transmissions are not frequent. However, this will lead to an unacceptable high loss rate of the communication tasks when the transmissions are frequently triggered. In asynchronous mode, when the task to transmit, the scheduling will not immediately to process; however, the priority communication tasks are added to the queue in sequence, then the wireless sensor through state machine to fetch the head of the communication task in the queue, and executes the task scheduling function. This model greatly reduces the tasks' loss, thus determining the wireless sensor network (WSN) which is formed in one of the biggest communication task captains that are of great help to guide sensor network design.  Figure 4 shows *n*-task communication scheduling based on the queuing theory model in the wireless sensor network.

Input process: communication tasks are divided into *N* levels. The first level has the highest priority, the second priority has secondary priority, and so forth. The *N* level has the lowest priority. Assume that each communication task interval is the Poisson distribution or negative exponential distribution. The average time of the interval of the *i*th level communication task is *λ*_*i*_.

Queuing rules: the task responses as soon as the communication task arrival by the background task execution call service, when the task is not scheduling executed in the system. The high-priority communication tasks priority is executed in sequence until the end of all high-priority tasks in the queue. When the low-priority tasks are executing, the high-priority task takes over the low-priority task and the low-priority task will return to the queue, the same priority communication task followed by FCFS rues.

Service process: wireless sensor network uses the state machine to drive communication task process, assuming that each time of the communication task is exponential distribution, and the average service of level *i* communication task rate is *μ*_*i*_.

The WSN communication tasks queue performance parameters [21]: (a) absolute throughput *A* is the average time of task service in the unit time; (b) relative throughput *Q* is the ratio of all the severed tasks and all request tasks in the unit time; (c) the queue length of the average value *L*_*s*_ is all communication tasks in the WSN; (d) the queue of average length *L*_*q*_ is the average number of the waiting tasks in the queue; (e) the average sojourn times *W*_*s*_ are the average of the task waiting for service time *W*_*q*_ and the average service time *τ* (then *W*_*s*_ = *W*_*q*_ + *τ*); (f) busy period *T*_*b*_ is the random parameter; (g) system loss rates *P*_loss_ are the overflow probability.

## 5. Priority Queue with Two Classes of Tasks

**Figure 5:**
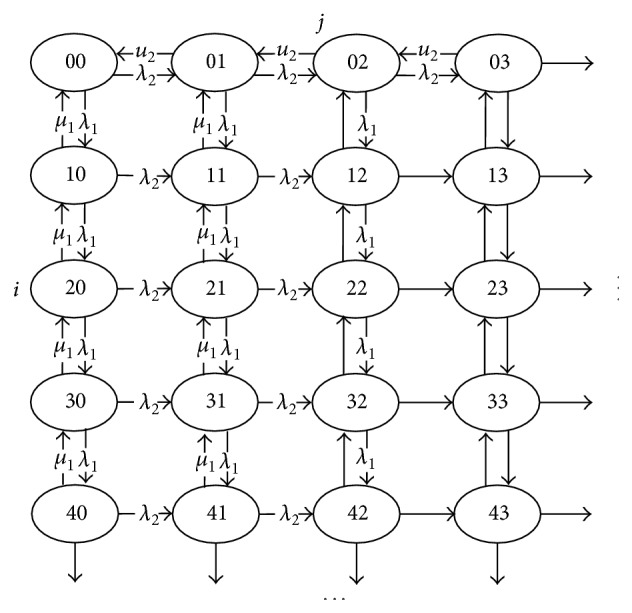
System state spaces and the transfer process.

Assume the queuing system is the preemptive priority. The *i*th task arrival is Poisson distribution with parameter *λ*_*i*_; the service time is exponentially distributed with parameter *μ*(*i* = 1,2,…). Level 1th priority communication task is more priority than level 2th priority task. The system state is *E* = {(*i*, *j*); 0 ⩽ *i*, 0 ⩽ *j*}, *i*(*j*) represents the 1(2) level of the communication tasks. The system state space distribution *P*(*i*, *j*) = {*P*_*i*,*j*_, 0 ⩽ *i*, 0 ⩽ *j*}. The system transition process is depicted in Figure 5.

## 6. Tasks Performance Indicators in Sensor Network

**Figure 6:**
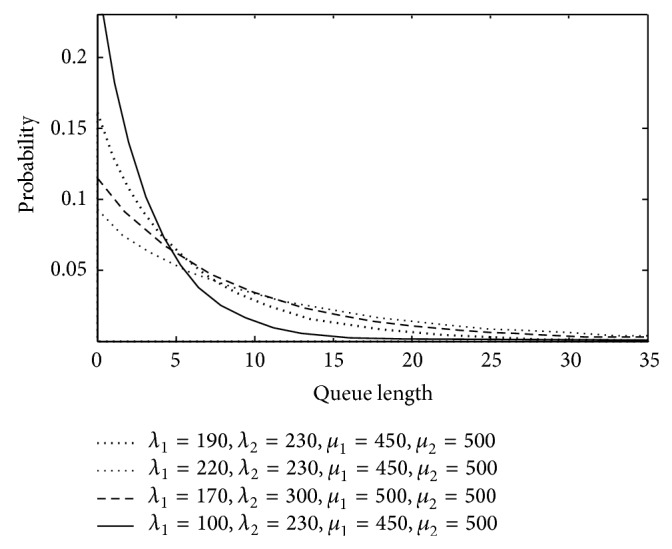
Probability and queue length graph.

The priority tasks processing is as follows: when the 1st level task with parameters *λ*_1_ and the 2nd level task with parameters *λ*_2_ arrive, service times of two level tasks are the *μ*_1_ and *μ*_2_, the task's priority is reduced in sequence, and they share the waiting queue. The recursive calculation of probability matrix {*P*_*i*,*j*_, 0 ⩽ *j* ⩽ *N*} needs a large amount of calculation; therefore, Matlab software is the necessary software. The M/M/1 preemptive priority queue model is used in the experiments. The arrival of the *i*th level task process as a Poisson distribution with the parameter *λ*_*i*_ and service time as a negative exponential distribution with the parameter *μ*_*i*_  (*i* = 1, 2). The 1st level tasks are more priority than the 2nd level communication tasks. Assume the parameters are *λ*_1_ = 170, *λ*_2_ = 300, *μ*_1_ = 500, and *μ*_2_ = 700.

(*1) Steady-State Queue Length*.  Figure 6 shows the probability and the queue length as the *μ*_2_ increasing. The vertical axis is the probability, and the horizontal axis is the queue length. In the probability matrix, *P* = 0; the maximum queue length can be obtained in the system and assures that task's buffer is enough to calculate the task's loss.

In the simulation, *L*_*s*_ = 40, which is the maximum queue length; when *P* = 0, the arrivals of the task's probability are 0; this means the possibility of task arrival does not exist, and the length does not grow. Then, when *P* = 0, the length can be regarded as the largest queue length.

(*2) Average Sojourn Time*. Assume that every level of task arrival is Poisson distribution. The *i*th level tasks are with the parameter *λ*_*i*_, the service time is the negative exponential distribution, and the average service time is 1/*μ*. The average sojourn times *W*_*s*1_ and *W*_*s*2_ are calculated in the following formulas:

(1) ws1=1μ−λ1,wq1=ws1−1μ=λ1μ(μ−λ1),

(2) ws1=(1+λ1λ2)×(1μ−λ1−λ2)−λ1λ2×1μ−λ1,wq2=ws2−1μ.

(*3)* *Average Waiting Time* [22]. The same way, the queue of the average waiting times *W*_*q*1_ and *W*_*q*2_ is calculated as formula (1).

(*4) Wireless Sensor Usage Rate* [23]. *P* is the probability of the wireless sensor being idle; then *ρ* = 1 − *P*, where *ρ* is the occupancy probability of communication task [24, 25]. The greater *ρ* is, the greater the occupancy probability is. *ρ* is the service capacity or the load capacity. To consider the practicality of the model, the queue length is not unlimited. To determine the performance indicators, we take the queuing model of M/M/1/N and set the buffer capacity which is *m*.

(*5) Task's Throughput.* The communication task's time *T* is divide into three parts: they are the task's processing time *T*_*i*_, the state machine processing time *T*_*s*_, and the wireless sensor idle time *T*_*r*_, *T*_*i*_ + *T*_*s*_ ≈ *T*. The task's processing is more priority than the state machine processing. Assume the tasks service strength is *ρ*_*i*_; then the tasks processing time is *T*_*i*_ = *ρ*_*i*_ *T*, and the state machine processing time is *T*_*s*_ = (1 − *ρ*_*i*_)*T*.

The processing of the finite automata is the queuing model of M/M/1/N; the input processing with the parameter *λ*_*s*_ and the service time with the parameter *μ*_*s*_ are the negative exponential distribution; length of the buffer is *n*. The processing speed of the communication task is faster than the processing speed of the state machine. Thus, the actual processing capability of the state machine is *μ*_*sr*_ = (1 − *ρ*_*i*_)*μ*_*s*_, and the buffer length is *m*; then the loss rate is

(3) $$\begin{array}{c} P_{\text{lost}}=\frac{1-\rho_{sr}}{1-\rho_{sr}^{m+1}}\rho_{sr}^{m}. \end{array}$$

In particular, *ρ*_*sr*_ = *λ*_*s*_/*μ*_*sr*_.

As to formula (1), the calculation which the state machine throughput computes is the following formula:

(4) $$\begin{array}{c} \lambda_{0}=\lambda_{s}\left(1-\frac{1-\rho_{sr}}{1-\rho_{sr}^{m+1}}\rho_{sr}^{m}\right). \end{array}$$

When the sensor network is severely overloading, the *λ*_*s*_ ≫ *μ*_*sr*_, and the *ρ*_*sr*_ ≫ 1. As to formula (2), formula (5) is calculated, due to formula (5), and the speed of the parameter *λ*_*i*_ affects the performance of the task processing; the greater *λ*_*i*_ is, the lower the performance of the task processing is. When the communication tasks processing rate *μ*_*i*_ and the state machine processing rate *μ*_*r*_ remain unchanged, the performance curve is a straight line in which the slope is −*μ*_*r*_/*μ*_*i*_. Consider

(5) $$\begin{array}{c} \lambda_{0}\approx \mu_{sr}=\left(1-\rho_{i}\right)\mu_{r}=\left(1-\frac{\lambda_{i}}{\mu_{i}}\right)\mu_{r}. \end{array}$$

*(6) Wireless Sensor Processing Capacity.* Assume the processing capacity is of *N* mips; the quantity of the tasks which need to be executed in the task's processing is *C*_1_ and the quantity of the tasks which need to be executed in the state machine is *C*_2_; then the task's processing capacity computes as formula (3); formula (6) is as follows:

(6) ρsr=λsμsr=λi(1−ρi)μs=λi(1−(λi/μi))μs=λi(1−(λi/(N/C1)))(N/C2)=λiC2N−λiC1.

Take formula (6) into formula (3); the loss rate *Q* is

(7) Q=Plost=1−(λiC2/(N−λiC1))1−[λiC2/(N−λiC1)]m+1×(λiC2)m[N−λiC1]m=(λiC2)m∑i=0m[N−λiC1]i(λiC2)m−i.

Formula (7) is the relationship between the loss rate and the processing capacity. It helps to determine the requirements of the task's processing.

## 7. Experiment

### 7.1. Experiment Set

We evaluated our matching approach by regular expression sets extracted from two real-world systems named L7-Filter and Snort. L7-Filter is famous open source application layer traffic classier for Linux. The payload content of a flow and identified its application level protocol are reassembled through regular expression matching. Snort is a well-known open-source intrusion detection system, which can be configured to perform protocol analysis, probes, and content inspecting over online traffic by detecting a variety of worms. Two sets are chosen as *R* = *r*_1_; *r*_2_; …; *r*_*n*_ to perform the experiments. The experiment parameters, ES, MP, are set, as shown in Table 1. Then, the local optimal value can be obtained during our experiments.

Print size denotes the memory occupation of prints after the profiteering stage. Group number is the group number of correlative regular expression according to the parameter *γ*. Average similarity is the average value of similarity in each group (see (2)). Package size is the testing packages length. Our simulation environment is based on NS-2 (Network Simulator, version 2). We set the number of nodes from 20 to 100. Number of suspicious package is the number of package that needs to be verified after the profiteering stage. Lastly, we calculate our experiment efficiency by their average executing cost: Efficiency = (Num of suspicious package/Total package Number) × (*F*(*g*_*i*_)/Group Number).

**Figure 7:**
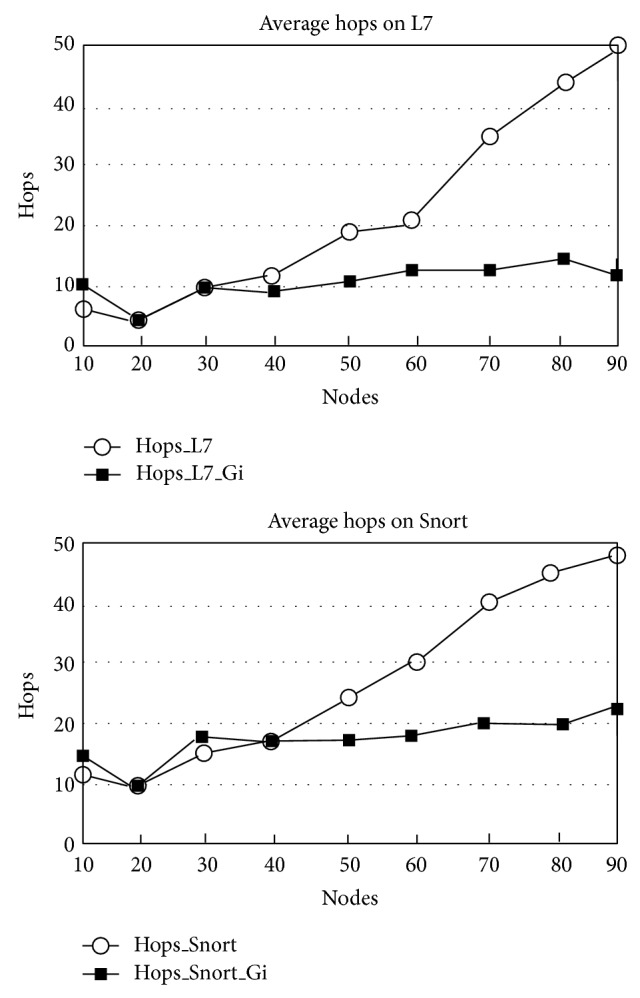
Results of node average hops in NS-2.

Table 2 demonstrates that we can get a good efficiency promotion from 73.17% to 89.73%. A regular matching comparison was performed with our strategy and normal approach. The average hop and average *F*(*G*_*i*_) variation tendency and the number of nodes are shown in Figure 7. L7-Filter and Snort were tested separately. *y*-axis is hops and *x*-axis indicates the nodes number. From the results figure, a significant difference can be observed: when nodes are more than 30, the hops number of using *G*_*i*_ decreases sharply.

Figure 7 demonstrates that the matching approach can test and verify the packages efficiently when the number of wireless nodes is more than 30, which indicates that our approach can be well adapted to medium or large scale distributed wireless sensor network. On the other hand, there is no major difference in average hops when the system is handling a small group of wireless nodes. Comparing with other end-to-end strategies [9], our approach provides a well scalable way to construct intrusion detection system by integrating distributed wireless sensor nodes. Based on appropriate parameters, network attacks can be monitored by our system in an effective way.

### 7.2. Experiment of Queue Model

The experiment compares two groups of the performance results, which computed by the queuing theory and got the results from the software NS2. The NS2 platform simulated the STM32W108 sensor networking; we found that it is affected with the following parameters: the average length of stay for communication tasks, the average queue waiting time of tasks, the occupancy rate, and the task throughput.

Simulation software configuration communication tasks take the high-priority traffic and low-priority communications task two categories. Design the task's scheduling function and assure *C*_1_ is approximately 400 operations and *C*_2_ is about 4000 operations. The program triggers communication according to the different experimental parameters *λ* and *m* to test the influence on wireless sensor performance. The experiment of the results compares with calculation results of the method based on queuing theory to verify the creditability of the method. The statistics are shown in Table 3.

When the sensor overloaded, the sensor network handles the task for a long time, and the state machine processing has no support to the sensor network. The actual speed of sensor processing *μ*_*sr*_ is far less than *μ*_*s*_. When the wireless sensor network needed specific requirements *t* of the loss rate and the sensor throughput, it can take the speed of the scheduling to the requirements.

For formula (7), it can understand the relationship between the loss rate and the capacity of scheduling. It can easily choose the right sensor in the network which will greatly improve the quality of service.

### 7.3. The Methods of Analysis

The previous method of finite automata has discussed the regular expression matching system security in WSN, and the evaluation of the performance is ignored to research. The matching method in some extent can maintain the precision and accuracy in some systems; it can be used in a specific environment; the performance of the evaluation in the wireless sensor network (WSN) that we had researched is in the universal environment; it is necessary to consider the problem of the restrictions, such as the capacity of the buffer, the length of the queue in the processing time, the performance of the calculation that needs enough buffer for the task processing, and the work conditions. In our research, the approach which took the matching method and the evaluating performance together is a new topic. The method ensures maintaining the system security, reducing the loss rate of the communication task, and improving the accuracy of the schedule. The universal approach can be used in lots of environments; in the wireless sensor network, the approach has a good, excellent performance.

## 8. Conclusions

This paper presented a regular expression matching approach for the wireless sensor network security systems, which is proposed to take the advantage of sensor nodes collaboratively, which divides the matching into matching preprocessing phase, validation phase, and performance evaluation phase. This method is based on queuing model to evaluate the performance of scheduling for the wireless sensor network. The experimental results show that our approach can speed up the efficiency of regular expression by at least 71% for the regular expression set by Snort and L7-Filter systems. And the queue model helps to obtain the communication tasks probability distribution and the relation between the task's processing capability and the task's response time, sensor throughput, and so forth.

The future work will focus on the following two aspects: the work will also extend the proposed approach and explore its feasibility for other network areas and continue to improve the queuing model to make it closer to the real wireless sensor which will raise the accuracy of the model.

## Figures and Tables

**Table 1:** Experimental parameters setting.

Parameter	L7-Filter	Snort
Num. of RegExp	161	166
Num. of DFA states	1432	1257
*β* (expression size)	256	256
ψ (relevance frequency)	[0.23, 0.34]	[0.23, 0.34]
*η* (match probability)	[0.75, 0.99]	[0.75, 0.99]
*γ* (similarity)	[0.5, 10]	[0.5, 10]

**Table 2:** Experimental data.

	Parameters					
Results	L7-Filter			Snort		
	*η*_1_ = 0.24, *γ*_2_ = 0.77, *ψ*_1_ = 0.6	*η*_2_ = 0.26, *γ*_2_ = 0.83, *ψ*_2_ = 5	*η*_3_ = 0.36, *γ*_3_ = 0.98, *ψ*_3_ = 10	*η*_1_ = 0.24, *γ*_2_ = 0.77, *ψ*_1_ = 0.6	*η*_2_ = 0.26, *γ*_2_ = 0.83, *ψ*_2_ = 5	*η*_3_ = 0.36, *γ*_3_ = 0.98, *ψ*_3_ = 10
Print size	0.15 MB	0.31 MB	0.12 MB	0.25 MB	0.39 MB	0.24 MB
Group number	14	17	11	14	15	12
Average similarity	0.83	0.98	0.95	0.89	0.89	0.97
Package size	1024 B	2048 B	4096 B	1024 B	2048 B	4096 B
Number of suspicious package	253	83	122	41	114	48
Efficiency	89.73%	73.17%	76.5%	83.1%	77.94%	85.35%

**Table 3:** Experimental data statistics.

	Parameters			
Indicator	Queuing theory model calculation results		NS2 simulation results	
	*λ*_1_ = 180, *λ*_2_ = 270, *μ*_1_ = *μ*_2_ = 700, *m* = 100	*λ*_1_ = 150, *λ*_2_ = 400, *μ*_1_ = *μ*_2_ = 700, *m* = 35	*λ*_1_ = 180, *λ*_2_ = 400, *m* = 100	*λ*_1_ = 150, *λ*_2_ = 400, *m* = 35
Queue length	120	40	112	38
Average sojourn time(s)	*W*_*s*1_ = 0.0019230 *W*_*s*2_ = 0.005385	*W*_*s*1_ = 0.0018182 *W*_*s*2_ = 0.008485	*W*_*s*1_ = 0.0018240 *W*_*s*2_ = 0.005062	*W*_*s*1_ = 0.0016401 *W*_*s*2_ = 0.00849
Average waiting time(s)	*W*_*q*1_ = 0.0004945 *W*_*q*2_ = 0.004956	*W*_*q*1_ = 0.0003924 *W*_*q*2_ = 0.0069565	*W*_*q*1_ = 0.0003982 *W*_*q*2_ = 0.0045412	*W*_*q*1_ = 0.0002116 *W*_*q*2_ = 0.006783
Wireless sensor usage rate	95.5%	81.9%	96.6%	88.3%
Sensor network throughput	436.000	474.300	453.954	423.561
Tasks loss rate	2.70677%	0.099%	3.77%	0.098%
